# PEX16 contributes to peroxisome maintenance by constantly trafficking PEX3 via the ER

**DOI:** 10.1242/jcs.146282

**Published:** 2014-09-01

**Authors:** Alexander Aranovich, Rong Hua, Andrew D. Rutenberg, Peter K. Kim

**Affiliations:** 1 Program in Cell Biology, Hospital for Sick Children, Toronto, ON M5G 0A4, Canada; 2 Department of Biochemistry, University of Toronto, Toronto, ON M5S 1A8, Canada; 3 Department of Physics and Atmospheric Science, Dalhousie University, Halifax, NS B3H 1Z9, Canada

**Keywords:** ER, Organelle biogenesis, Peroxisomes, Live cell imaging, Membrane trafficking, Protein trafficking

## Abstract

The endoplasmic reticulum (ER) is required for the *de novo* biogenesis of peroxisomes in mammalian cells. However, its role in peroxisome maintenance is unclear. To explore ER involvement in the maintenance of peroxisomes, we redirect a peroxisomal membrane protein (PMP), PEX3, to directly target to the ER using the N-terminal ER signal sequence from preprolactin. Using biochemical techniques and fluorescent imaging, we find that ER-targeting PEX3 (ssPEX3) is continuously imported into pre-existing peroxisomes. This suggests that the ER constitutively provides membrane proteins and associated lipids to pre-existing peroxisomes. Using quantitative time-lapse live-cell fluorescence microscopy applied to cells that were either depleted of or exogenously expressing PEX16, we find that PEX16 mediates the peroxisomal trafficking of two distinct peroxisomal membrane proteins, PEX3 and PMP34, via the ER. These results not only provide insight into peroxisome maintenance and PMP trafficking in mammalian cells but also highlight important similarities and differences in the mechanisms of PMP import between the mammalian and yeast systems.

## INTRODUCTION

Peroxisomes are metabolic organelles found in almost all eukaryotic cells. In the mammalian system, they are required for the metabolism of very long chain fatty acids, purines, polyamines and hydrogen peroxides in addition to the biosynthesis of key components, such as plasmalogens and bile acids (van den Bosch et al., 1992). Defects in any of the peroxisome biogenesis genes, called peroxins (PEX), result in a group of hereditable genetic diseases called peroxisome biogenesis disorders (PBDs) (Steinberg et al., 2006).

Studies on PBDs and the use of yeast genetics have revealed the protein import mechanisms of peroxisomes to be distinct from other single-membrane-bound organelles. Like mitochondria and chloroplasts, peroxisomes are able to import all of their matrix (lumen) proteins directly from the cytosol (Ma et al., 2011; Rucktäschel et al., 2011). However, peroxisomal membrane proteins (PMPs) can be imported via two distinct pathways. The group I import pathway targets newly synthesized PMPs to the endoplasmic reticulum (ER) before routing them to peroxisomes, whereas group II PMPs are imported directly into peroxisomes from the cytosol (South et al., 2001; Titorenko and Rachubinski, 2001).

Whereas the group I pathway is necessary for *de novo* biogenesis of peroxisomes from the ER in cells without pre-existing peroxisomes, conflicting evidence exists on the extent that these two pathways are used in normal cells (Theodoulou et al., 2013). Using both fluorescent imaging and biochemical techniques, PMPs in the yeast *Saccharomyces cerevisiae* have been shown to target to the ER in conditions where there are no pre-existing peroxisomes, whereas in normal cells, they appear to be imported directly to peroxisomes (Motley and Hettema, 2007). Similarly, after cell division in *S. cerevisiae*, a key peroxisome biogenesis factor Pex3p appears to be directly imported to pre-existing peroxisomes (Menendez-Benito et al., 2013). These results apparently conflict with a recent demonstration that most PMPs in *S. cerevisiae* can be targeted directly to the ER via the post-translational import system, which suggests that most PMPs use the group I import pathway, even in *S. cerevisiae* with pre-existing peroxisomes (van der Zand et al., 2010; Thoms et al., 2012).

Similar conflicting results have also been reported in mammalian systems. There, PEX16, an essential PMP involved in peroxisome biogenesis, is targeted to the ER before it is transported to peroxisomes (Kim et al., 2006). Nevertheless, based on colocalization and *in vitro* targeting assays, others have argued that mammalian PMPs only target to peroxisomes via the group I pathway in cells without pre-existing peroxisomes, and that the ER does not contribute to the maintenance of mammalian peroxisomes (Matsuzaki and Fujiki, 2008; Huybrechts et al., 2009). Rather, it has been suggested that all PMPs in normal cells are targeted directly to peroxisomes without accessing the ER (Lazarow and Fujiki, 1985; Sacksteder et al., 2000; Matsuzaki and Fujiki, 2008; Huybrechts et al., 2009; Schmidt et al., 2012).

We believe that the role of the ER in targeting PMPs to pre-existing peroxisomes has been erroneously discounted owing to the difficulty in detecting PMPs in the ER at steady state. Rather than being completely absent from the ER, PMPs may be rapidly exported from the ER to peroxisomes resulting in their short time of residence in the ER (Nuttall et al., 2011; Schmidt et al., 2012). To test this hypothesis, we have developed a biophysical imaging technique to quantify the kinetics of PMP import into peroxisomes. With the assumption that import rates of PMPs that are directly imported to peroxisomes from the cytosol will differ from those routed through the ER, quantification of import rates of various PMPs provides a method to determine whether multiple pathways of PMP import into peroxisomes exist. We report here that the PMPs explored are imported into peroxisomes at two distinct rates: a faster import rate similar to matrix proteins (group II pathway) and a slower rate similar to that of a PMP forced into the group I pathway. We find that PEX16 is imported into peroxisomes via the group I pathway, and might also play a direct role in regulating this pathway. Furthermore, we present evidence that the group I pathway may be the default route to peroxisomes for all PMPs. Based on these results, we propose a model for the mammalian PMP import system in which the ER constitutively provides both lipids and proteins for the maintenance of pre-existing mature peroxisomes.

## RESULTS

### ER-targeting PEX3 is routed to peroxisomes via the ER

**Fig. 1.**
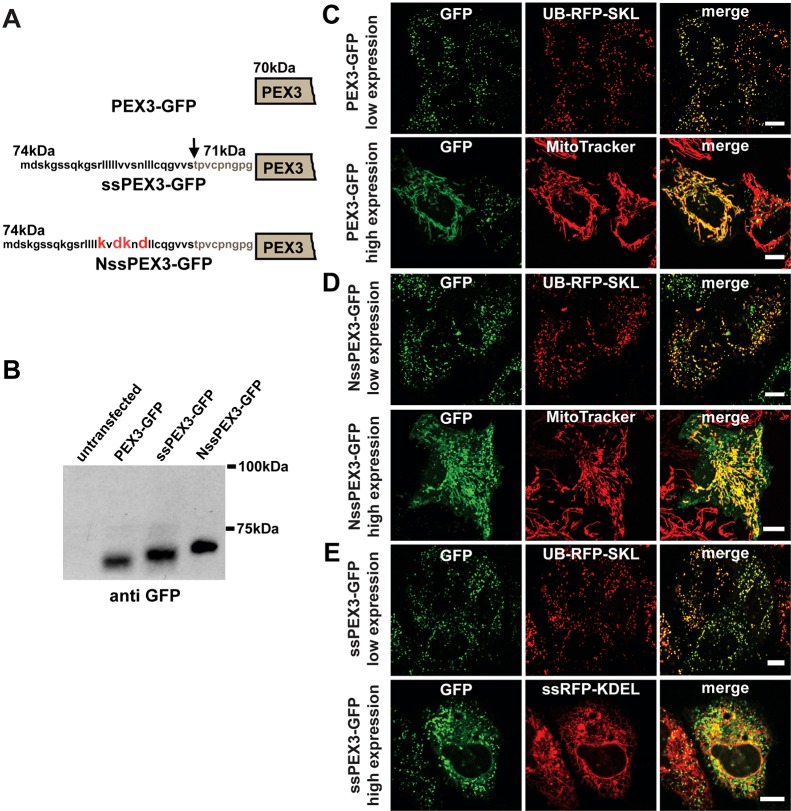
**ssPEX3-GFP protein is targeted to peroxisomes via the ER.** (A) Representation of N-terminal ends of PEX3-GFP, ssPEX3-GFP and NssPEX3-GFP. GFP tagged at the C-terminus of each PEX3 construct is not shown. The amino acid sequences correspond to the signal sequence of preprolactin. The cleaved portion of the signal sequence is shown in bold, the arrow indicates the cleavage site. The calculated molecular mass of each construct, including the cleaved form of ssPEX3-GFP, is given in kDa. Mutations within the signal sequence that abolish the ER-targeting function of the signal sequence are highlighted in red for NssPEX3-GFP. (B) Signal sequence cleavage assay. Cells expressing PEX3-GFP, ssPEX3-GFP or NssPEX3-GFP were lysed 20 hours after transfection and analyzed by western blotting using an anti-GFP antibody. (C–E) Analysis of the subcellular localization of each PEX3 construct exogenously expressed in HeLa cells by using fluorescent live-cell microscopy 20 hours after transfection. Representative confocal fluorescence microscopy images for PEX3-GFP (C), NssPEX3-GFP (D) and ssPEX3-GFP (E) of both low and high expression levels are shown. For cells that expressed PEX3 at low levels, co-expression with the peroxisomal marker UB-RFP-SKL is shown. In images showing cells that express PEX3 at high levels, cells were stained with either MitoTracker Red (C,D) or they show co-expression of PEX3 with ER targeting ss-RFP-KDEL (E). See supplementary material Fig. S1 for magnification of high ssPEX3-GFP. All images were taken at the same settings and brightness for each panel was enhanced equally for presentation. Scale bars: 10 µm.

It is not clear whether the ER is involved in the maintenance of peroxisomes in normal mammalian cells with pre-existing peroxisomes. To determine whether such cells can transport peroxisomal membrane proteins (PMPs) to peroxisomes via the ER (i.e. the group I PMP pathway), we designed a PMP that is ‘forced’ to target to the ER co-translationally. Previously, PEX3 tagged with an ER-targeting signal sequence was shown to complement a PEX3-deficient cell line that lacked peroxisomes, suggesting that ER-localized PEX3 can form peroxisomes (Toro et al., 2009). Using a similar construct, we asked whether an ER-localized PEX3 could be transported to pre-existing functional peroxisomes. The ER-targeting PEX3, named ssPEX3-GFP, consists of PEX3 with a cleavable ER-targeting signal sequence from bovine preprolactin at its N-terminus and monomerized enhanced GFP (EGFP) at its C-terminal end (Fig. 1A). The preprolactin signal sequence was selected, as it is a well-characterized signal sequence that has a high ER-targeting fidelity (Jungnickel and Rapoport, 1995).

To assess whether ssPEX3-GFP is targeted to the ER, we examined for the cleavage of the signal sequence portion of ssPEX3-GFP, which is cleaved by the ER signal sequence peptidase inside the ER lumen during translation-coupled translocation into the ER (Jungnickel and Rapoport, 1995). The cleavage of the signal sequence in ssPEX3-GFP expressed in HeLa cells was determined by comparing its molecular mass with that of PEX3-GFP (without a signal sequence) and that of NssPEX3-GFP (with a nonsense signal sequence) by using SDS-PAGE (Fig. 1A). The NssPEX3-GFP construct is similar to ssPEX3-GFP, except for four mutations within the signal sequence that make it less hydrophobic and, therefore, unrecognizable by the signal recognition particle that would target it to the ER (von Heijne, 1985). SDS-PAGE comparison of these three constructs showed a single band for ssPEX3-GFP with mobility between that of PEX3-GFP and NssPEX3-GFP (Fig. 1B). This was consistent with cleavage at the predicted cleavage site (Fig. 1A). The presence of a single band confirmed that the majority of the synthesized proteins were targeted to the ER.

We next used confocal microscopy to examine the subcellular localization of the three PEX3 constructs transiently transfected into HeLa cells (Fig. 1C–E). UB-RFP-SKL was used to visualize pre-existing peroxisomes, ssRFP-KDEL for the ER and MitoTracker Red for the mitochondria. UB-RFP-SKL is a chimera of monomeric red fluorescent protein (mRFP) fused to ubiquitin (UB) at the N-terminus and the type 1 peroxisomal targeting signal (PTS1) tripeptide Ser-Lys-Leu (SKL), at the C-terminus (Kim et al., 2008). The ubiquitin motif in UB-RFP-SKL was used to minimize the accumulation of non-targeted protein in the cytosol by its degradation through the ubiquitin-proteasome degradation pathway. PEX3-GFP without an ER signal sequence localized mainly to peroxisomes as previously shown, but it was also found to localize to mitochondria in the few cells expressing PEX3-GFP at higher levels (Fig. 1C) (Soukupova et al., 1999; Sacksteder et al., 2000).

The subcellular localization of the non-ER-targeting NssPEX3-GFP was similar to PEX3-GFP. At low expression levels, it colocalized with the peroxisome marker UB-RFP-SKL; at higher expression levels, it was found on both peroxisomes and mitochondria (Fig. 1D). The localization of ssPEX3-GFP, however, differed from wild-type PEX3 and NssPEX3. As shown in the representative cells in Fig. 1E, at low expression levels ssPEX3-GFP localized almost exclusively to peroxisomes but, in the few cells with very high expression of ssPEX3-GFP, it was also found on the ER but not mitochondria (Fig. 1E; supplementary material Fig. S1). Because the majority of the signal sequence on ssPEX3-GFP was cleaved under similar transfection conditions (Fig. 1B), these results suggested that ssPEX3-GFP initially targets to the ER before routing to peroxisomes. When ssPEX3-GFP was expressed at high levels, its presence in the ER implied that the export of ssPEX3-GFP from the ER to peroxisomes is saturable. Similar ER accumulation has previously been reported for PEX16 (Kim et al., 2006).

### ssPEX3 targets to pre-existing peroxisomes

**Fig. 2.**
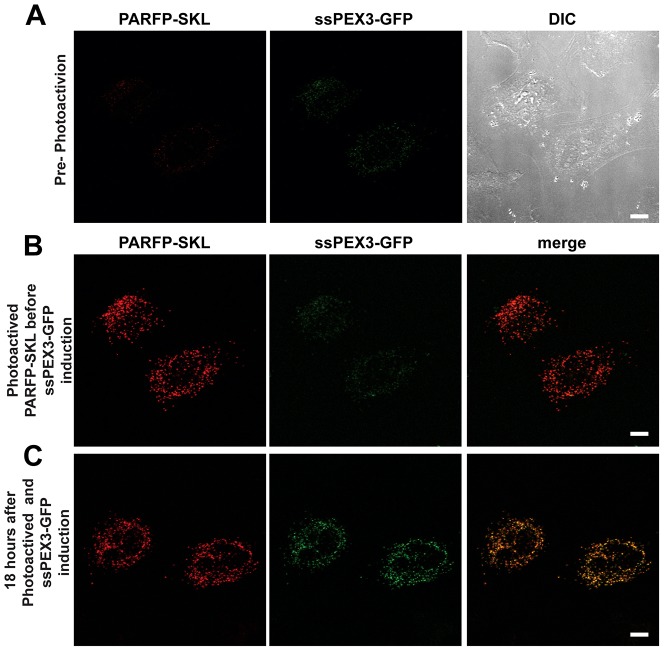
**ssPEX3-GFP targets to pre-existing peroxisomes.** HeLa Tet-On cells transfected with plasmids encoding ssPEX3-GFP under a TRE-Tight promoter and photoactivatable RFP-SKL (PARFP-SKL) under a CMV promoter. (A) Representative HeLa Tet-On cells before photoactivation of PARFP-SKL. Channels for PARFP-SKL, ssPEX3-GFP and DIC images are indicated. (B) Image of the same cell immediately after photoactivation of PARFP-SKL (red) followed by the addition of doxycycline to induce ssPEX3-GFP (green) expression (∼5 minutes). (C) The same cells as above 18 hours after photoactivation and induction. Scale bars: 10 µm.

Close examination of the images in Fig. 1 showed ssPEX3-GFP colocalized to all punctate structures positive for the matrix protein UB-RFP-SKL (Fig. 1E), which suggested that ssPEX3-GFP is targeted to pre-existing peroxisomes. To confirm that this is, indeed, the case, we transiently co-expressed a photoactivatable RFP with PTS1, PARFP-SKL, with a plasmid construct that placed ssPEX3-GFP under a tetracycline-response element inducible system. PARFP-SKL was photoactivated before ssPEX3-GFP expression was induced with doxycycline (Fig. 2A). Because PARFP-SKL was not re-photoactivated, all PARFP-SKL-positive puncta that existed before the induction of ssPEX3-GFP were considered pre-existing peroxisomes. After 18 hours of induction, ssPEX3-GFP were found colocalized with the photoactivated PARFP-SKL, demonstrating that ssPEX3-GFP was able to target to pre-existing peroxisomes (Fig. 2B,C).

### ssPEX3-GFP complements PEX3 deficiency

**Fig. 3.**
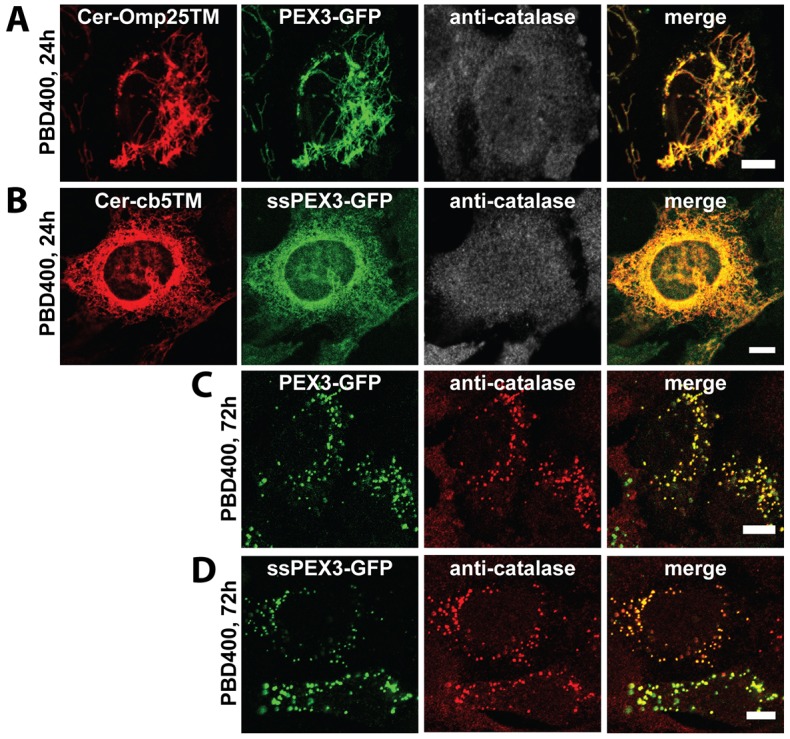
**ssPEX3-GFP and PEX3-GFP complement PEX3 deficiency in PBD400-T1 cells.** Representative immunofluorescence confocal images of PEX3-deficient PBD400-T1 cells transiently co-transfected with plasmids encoding PEX3-GFP and mCerulean-Omp25TM (a mitochondrial marker) (A) or ssPEX3-GFP and mCerulean-cb5TM (an ER marker) (B) and immunostained for endogenous catalase 24 hours (A,B) and 72 hours after transfection (C,D), as indicated. The analyses of 300 cells expressing the PEX3 constructs 72 hours after transfection from three independent experiments resulted in almost 100% peroxisome recovery. Scale bars: 10 µm.

Next, we asked whether the ER-targeting ssPEX3-GFP was functional by examining whether it can complement the PEX3-deficient human cell line PBD400-T1 (South et al., 2000). In the early stages of expression (24 hours after transfection), PEX3-GFP was found localized to mitochondria, whereas ssPEX3-GFP localized to the ER, suggesting a difference in their membrane-targeting mechanisms (Fig. 3A,B). As previously shown, complementing these cells with full-length PEX3 for 72 hours resulted in the formation of new peroxisomes as confirmed by the colocalization of PEX3-GFP with endogenous catalase in punctate structures (Fig. 3C) (South et al., 2000). Similarly, ssPEX3-GFP was able to complement PEX3 deficiency because its expression resulted in the formation of catalase-positive structures 72 hours after transfection (Fig. 3D).

### Quantification of peroxisomal import rate of PMPs

The observation that ssPEX3-GFP readily located to pre-existing peroxisomes suggested that the Group I PMP import pathway usually occurs in mammalian cells. We quantified the kinetics of PMP import into pre-existing mature peroxisomes to determine whether other PMPs also target to peroxisomes via the group I pathway. The ER trafficking pathway should be significantly slower than the direct import pathway owing to the inherent complexity of protein trafficking between two organelles (Lodish, 1988; Zaal et al., 1999). Thus, we assert that PMPs routed through the ER to peroxisomes (group I pathway) will manifest a slower import rate into peroxisomes compared to directly targeted group II PMPs or peroxisomal matrix proteins.

**Fig. 4.**
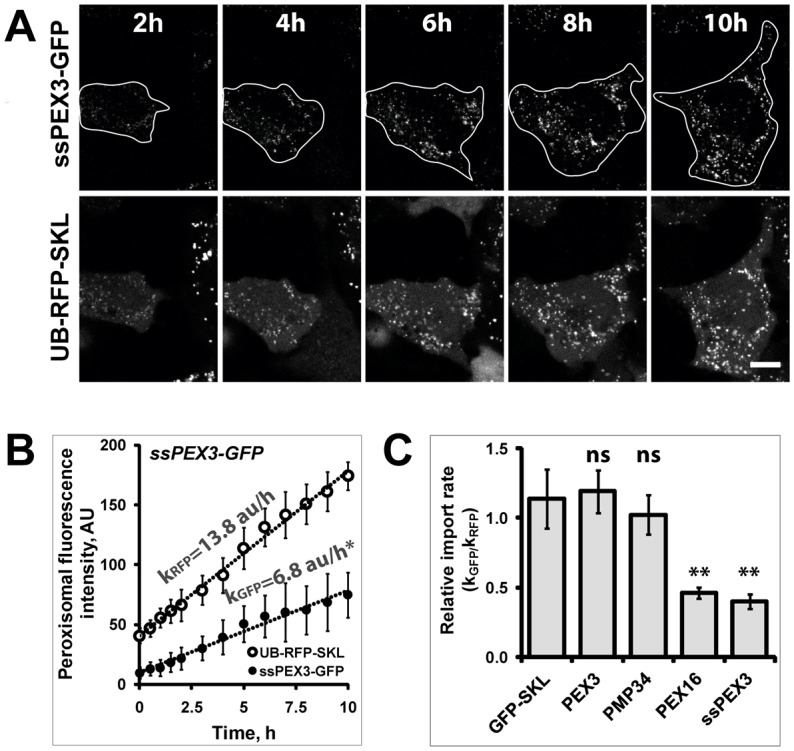
**PMP import kinetic quantification assay.** (A) Time-lapse imaging experiment of HeLa cells co-expressing ssPEX3-GFP with UB-RFP-SKL. GFP and RFP signals were acquired at 37°C over a period of 10 hours in CO_2_-independent medium in several different fields of view. Shown are representative time-lapse images at different time points. For presentation, the brightness of the images was enhanced equally for all frames. Scale bar: 10 µm. The boundaries of the analyzed cell are marked by white line on ssPEX3-GFP images. (B) The change in the fluorescent intensity within peroxisomes of ssPEX3-GFP (•) and UB-RFP-SKL (○) in the representative cell shown in A, is plotted against time to illustrate the rates of ssPEX3-GFP and UB-RFP-SKL import. The apparent peroxisome import kinetics is shown for both proteins. AU, arbitrary unit. Data were fit with linear regression (R^2^>0.98). Error bars indicate ±s.d. of signal between peroxisomes at a given time. Both the mean and the slopes were found to be significantly different (*P<0.001) by Student's *t*-test. (C) Summary for relative import rates of PEX3-GFP, PMP34-GFP, PEX16-GFP and ssPEX3-GFP (PEX3, PMP34, PEX16 and ssPEX3, respectively) respective to the import rate of UB-RFP-SKL (k_GFP_/k_RFP_) are shown in the bar graph. UB-GFP-SK (GFP-SKL) was used as a control. The analyses were performed on cells at early stages of protein expression. At least 17 cells from three independent experiments were analyzed for each GFP-protein construct. The error bars represent ±s.e.m. The *P* value was determined using Student's *t*-test. Both the relative import rates of PEX16 and ssPEX3 were significantly different from the GFP-SKL control (**P<0.005), whereas PEX3 and PMP34 showed no significant difference (ns). Scale bars: 10 µm.

We measured the increase in the fluorescent signal of various GFP chimera PMPs with respect to the artificial peroxisomal matrix protein UB-RFP-SKL by using time-lapse imaging of living cells (see Materials and Methods, supplementary material Figs S1–S3). PTS1-containing polypeptides are directly imported into mature import-competent peroxisomes from the cytosol (Santos et al., 1988). Fig. 4 shows the quantification of the apparent import rates of ssPEX3-GFP (k_GFP_) and UB-RFP-SKL (k_RFP_) into functional mature pre-existing peroxisomes using UB-RFP-SKL as a peroxisome marker. Time-lapse imaging was started at an early stage of the protein expression in order to analyze the apparent import rates of these proteins into peroxisomes (Fig. 4A). In the representative cell (Fig. 4A), GFP and RFP signals within peroxisomes were quantified and corrected for background (Fig. 4B).

For ssPEX3-GFP-expressing cells, the entire population of UB-RFP-SKL-positive punctate structures was observed, and shows an increase in both RFP and GFP signals over time (Fig. 4A,B), which suggests that ssPEX3-GFP is readily imported into pre-existing peroxisomes. The GFP (Fig. 4B, black circles) and RFP (Fig. 4B, white circles) fluorescent signals within peroxisomes were plotted against time. Both the ssPEX3-GFP and the UB-RFP-SKL import into peroxisomes show a linear increase over time (R^2^>0.98) (Fig. 4B).

### The import rate of PEX16 is similar to that of ssPEX3-GFP but distinct from other PMPs

Using this quantification technique, we also determined the relative import rates for three distinct PMPs (PMP34, PEX16 and PEX3), and then compared them to the relative rates of the ER-targeting ssPEX3 and the matrix protein UB-GFP-SKL. PMP34 was chosen because it is not required for peroxisome biogenesis and its membrane topology differs from the other two PMPs (Soukupova et al., 1999; Honsho et al., 2001; Honsho et al., 2002). PMP34 has six transmembrane domains (TMs) whose ends are both at the cytosolic side (Honsho and Fujiki, 2001). In contrast, PEX3 is a type I membrane protein with a single TM near its N-terminus, which places its N-terminal end inside the matrix of peroxisomes; PEX16 has been predicted to have two TMs that have both ends at the cytosolic side (Soukupova et al., 1999; Honsho et al., 2002). ssPEX3-GFP was used as a control protein as it traffics to peroxisomes via the ER; UB-GFP-SKL was used as a direct trafficking control (Fig. 4C).

To compare the import rates of these GFP-fused proteins, we calculated their rate of import relative to the rate of UB-RFP-SKL import (k_GFP_/k_RFP_; Fig. 4B). The relative import rates of PEX3-GFP and PMP34-GFP did not significantly differ from each other or from the matrix protein UB-GFP-SKL (Fig. 4C). In contrast, the relative import rates of the ER-targeting PEX3 constructs ssPEX3-GFP and PEX16-GFP were both less than half of the rates of UB-GFP-SKL and the other two PMPs (Fig. 4C). To ensure that the difference in rates of these various PMPs was not due to variation of the imaging parameters, we also performed the time-lapse image acquisition of cells expressing UB-GFP-SKL together with either PEX3-GFP or PEX16-GFP (supplementary material Fig. S2A), and of cells expressing UB-GFP-SKL together with either PMP34-GFP or ssPEX3-GFP (supplementary material Fig. S2B). This enabled direct comparison of the apparent import rates k_GFP_ and k_RFP_ given in arbitrary units per hour (AU/h) of PEX3-GFP to PEX16-GFP, and PMP34-GFP to ssPEX3-GFP. Similar to the relative import rates (k_GFP_/k_RFP_), we found that PEX16-GFP and ssPEX3-GFP import rates are approximately half of those for PEX3-GFP and PMP34-GFP, respectively (supplementary material Fig. S2A,B).

Next, we examined whether the difference in the rates was due to differences in the expression levels of the different PMP constructs. No correlation was found when the apparent rate for ssPEX3-GFP or PEX3-GFP was plotted against the non-peroxisomal (Cyto-ER) fluorescent signal within a corresponding cell (supplementary material Fig. S2C,D). These results suggest that the apparent import rate of GFP-fused protein into peroxisomes is independent of the protein expression level. Furthermore, the co-expression of PMP-GFP constructs did not affect the apparent import rate of UB-RFP-SKL into peroxisomes (supplementary material Fig. S2A,B), suggesting that the import of matrix proteins is independent of expression of the co-transfected PMPs.

Overall, these results show that the import rate of PEX16-GFP into pre-existing peroxisomes is significantly slower than that of PEX3-GFP and PMP34-GFP. Instead, the PEX16-GFP import rate is similar to that of the ER-targeting ssPEX3-GFP. These results suggest that the majority of exogenously expressed PEX16 is targeted to the ER before being transported to peroxisomes. In contrast, in our system, the majority of exogenously expressed PEX3 and the multi-TM protein, PMP34, are imported directly to pre-existing peroxisomes.

### ER-targeted ssPEX3-GFP does not complement PEX16 mutation

In mammalian cells, PEX16 is able to recruit PEX3 into both the ER and peroxisomal membranes (Kim et al., 2006; Matsuzaki and Fujiki, 2008). However, most yeast strains, such as *S. cerevisiae* and *H. polymorpha*, do not express a PEX16 homologue. Instead, the *S. cerevisiae* homologue PEX3p is targeted directly to the ER via the post-translational translocon complex Sec61–Sec62–Sec63 (Hoepfner et al., 2005; Kragt et al., 2005; Tam et al., 2005; van der Zand et al., 2010; Thoms et al., 2012). Therefore, it is possible that the sole function of PEX16 in the mammalian system is to import PEX3 into peroxisomal membranes. Because mammalian PEX3 is thought to be involved in the import of PMPs into peroxisomes, recruiting PEX3 to the ER might allow for the subsequent import of other PMPs. If this is the case, then targeting PEX3 to the ER by means other than PEX16 might be sufficient for *de novo* biogenesis of peroxisomes from the ER in cells deficient in PEX16 function.

**Fig. 5.**
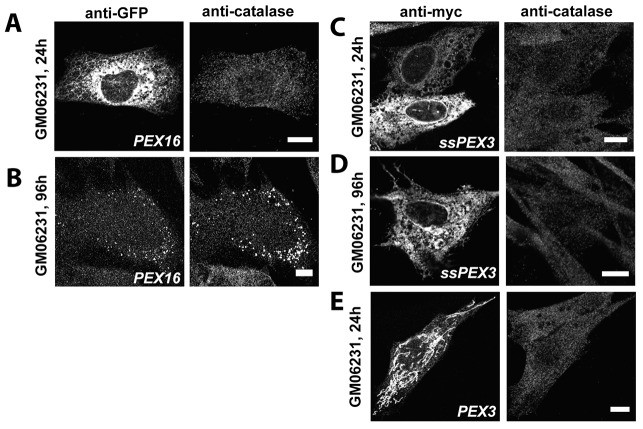
**Targeting of PEX3 to ER does not complement PEX16 deficient cells.** Representative immunofluorescent confocal images of the PEX16-deficient cell line GM06231, transiently transfected with plasmids encoding PEX16-GFP (A,B), ssPEX3×3myc (C,D) or PEX3×3myc (E). PEX16-GFP was found initially in the ER (A) at 24 hours; however, at 96 hours it was found colocalized with catalase-positive punctate structures suggesting complementation of PEX16 function (B). In contrast, ssPEX3×3myc is unable to complement PEX16 deficiency in GM06231 cells (C,D). ssPEX3×3myc is localized to the ER at 24 (C) and 96 (D) hours after transfection. No catalase-positive punctuate structures were detected. (E) PEX3×3myc is localized to mitochondria in GM06231 cells. Scale bars: 10 µm.

To test this hypothesis, we exogenously expressed ssPEX3-GFP in the peroxisome-free PEX16-deficient cell line GM06231 (Brocard et al., 2005). Given that ssPEX3-GFP is functional owing to its ability to complement a PEX3-mutant cell line (Fig. 3D), ssPEX3-GFP should be able to complement GM06231 cells if the sole function of PEX16 is to import PEX3 to ER membranes. For these experiments, we used the 3×Myc-tagged versions of ssPEX3 and PEX3 (ssPEX3-3×myc and PEX3-3×myc, respectively) to account for the unlikely possibility that the GFP motif would cause steric hindrance. The fact that PEX16-GFP can complement PEX16 function made it a suitable control (Fig. 5A,B). Similar to its localization in cells of the PBD400-T1 cell line, 24 hours after transfection PEX3-3×myc was found localized to mitochondria, whereas ssPEX3-3×myc was predominately found on the ER (Fig. 5C–E). In fact, the localization of ssPEX3-3×myc at 24 hours after transfection was similar to that of PEX16-GFP (Fig. 5A,C). However, unlike PEX16-GFP, which was able to complement the PEX16 mutation (Fig. 5B), ssPEX3-3×myc was not able to complement the PEX16 mutant cells even at 96 hours after transfection and remained localized to the ER (Fig. 5D). This result suggests that PEX16 has other function(s) in addition to its role as a receptor for PEX3 import.

### Knockdown of PEX16 slows down ssPEX3 transport from ER to peroxisomes

Another possible function of PEX16 could be its involvement in the process of trafficking PMPs from the ER to peroxisomes. To test this hypothesis, we quantified the import rates of several PMPs into pre-existing peroxisomes in cells depleted of *PEX16* expression by using RNA interference compared to control cells. If PEX16 is involved in the transport of PMPs from the ER to peroxisomes, then the amount and the rate of import of these PMPs should decrease in cells depleted of PEX16. Conversely, PMPs that are targeted directly to peroxisomes should not be affected.

**Fig. 6.**
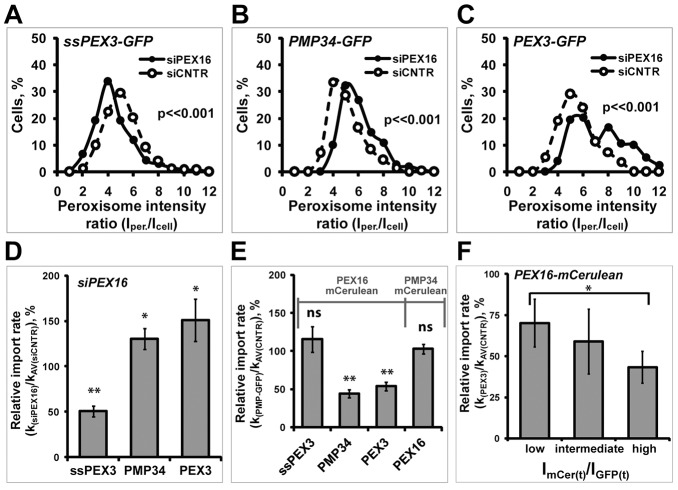
**Depletion of PEX16 expression delays ssPEX3-GFP import into peroxisome, whereas overexpression of PEX16 decreases the peroxisome import of PEX3-GFP and PMP34-GFP.** (A–C) The distribution of PMP-GFP signal within peroxisomes (I_per._) with respect to the average fluorescence signal, within the whole cell (I_cell_) treated with either siPEX16 or siCNTR, are shown in a histogram as a ratio (I_per._/I_cell_) for (A) ssPEX3-GFP, (B) PMP34-GFP, and (C) PEX3-GFP. The distributions for each PMP in siPEX16-treated cells were significantly different from that in control cells (siCNTR) (*P*<<0.001, *n* = 50). A scatter plot of the same data (A–C) is shown in supplementary material Fig. S3 (C–E). (D) The peroxisomal import rates [k_(siPEX16)_] of ssPEX3-GFP, PMP34-GFP, and PEX3-GFP in PEX16-depleted cells compared to siCNTR-treated [k_AVsiCNTR)_] cells are shown in a bar graph. (E) The peroxisomal import rates [k_(PMP-GFP)_] of ssPEX3-GFP, PMP34-GFP, PEX3-GFP or PEX16-GFP in cells co-expressing PEX16-mCerulean or PMP34-mCerulean, as indicated, compared to the average rates of the corresponding PMP-GFP construct in cells co-expressing mCerulean [k_AV(CNTR)_] are shown in a bar graph. (F) The rate of PEX3-GFP import into peroxisomes decreased with increasing PEX16-mCerulean expression. Shown here is a different representation of the data presented in E. Here, the relative import rate of PEX3-GFP compared to control is plotted based on its ratio of PEX16-mCerulean over the total PEX3-GFP fluorescence signal [I_mCer(t)_/I_GFP(t)_]. The rates are subdivided into three groups (with a comparable number of cells in each group) from low to high I_mCer(t)_ to I_GFP(t)_ ratio. The error bars indicate ±s.e.m. *P<0.05; **P<0.005; ns, not significant.

To deplete PEX16 protein levels, cells were treated with small interfering RNA (siRNA) targeting *PEX16* (siPEX16) or with non-targeting RNA (siCNTR) as the negative control. The depletion of *PEX16* mRNA was verified by quantifying *PEX16* mRNA levels using qRT-PCR (supplementary material Fig. S3A). For these experiments, we employed HeLa cells stably expressing RFP-SKL because it allowed for easier identification of pre-existing peroxisomes. To compare the distribution of PMPs within peroxisomal and non-peroxisomal compartments under various cellular conditions, the relative amount of PMPs in peroxisomes was determined by dividing the average fluorescence intensity of PMP-GFP within peroxisomes (I_per._) by the average fluorescence intensity within the whole cell (I_cell_) 20 hours after transfection. In this assay, a higher I_per._:I_cell_ ratio indicates a larger fraction of PMPs in peroxisomes. The distribution of the relative amount of PMPs in peroxisomes of 150 cells from three independent experiments are summarized as a histogram for cells treated with siPEX16 or siCNTR (Fig. 6A–C; supplementary material Fig. S3C–E). These results illustrate a significant decrease in the amount of ssPEX3-GFP within peroxisomes in cells depleted of endogenous PEX16 compared to control cells (*P*<<0.001; Fig. 6A), suggesting that depletion of PEX16 disrupts the localization of ssPEX3-GFP into peroxisomes. This decrease is probably not due to off-target effects that PEX16 siRNA might have on the cell, because co-expression of an siRNA-resistant PEX16 (PEX16*-mCerulean) was able to rescue the phenotype (supplementary material Fig. S3B). Unexpectedly, siPEX16-treated cells showed significantly stronger relative PMP34-GFP and PEX3-GFP signals within peroxisomes compared to control (*P*<<0.001; Fig. 6B,C), suggesting an increase of these protein fractions in peroxisomes.

To test whether the difference in the amount of PMPs in peroxisomes in siPEX16 versus siCNTR-treated cells is due to changes in their peroxisomal import rates, we compared the apparent import rate of PMP-GFP into peroxisomes in siPEX16 (k_siPEX16_)-treated cells against that of siCNTR-treated cells [k_AV(siCNTR)_]. For these experiments, siCNTR- and siPEX16-treated cells were imaged in parallel to allow for direct comparison between the two conditions (Fig. 6). In agreement with the distribution data for ssPEX3-GFP within peroxisomes (Fig. 6A), PEX16 depletion resulted in a significant decrease in the rate of ssPEX3-GFP import into pre-existing peroxisomes compared to control cells. This lower rate for ssPEX3-GFP in the PEX16 knockdown cells was not due to a difference in peroxisome numbers because depletion of PEX16 expression did not change the number of peroxisomes (supplementary material Fig. S3F). Because ssPEX3-GFP is targeted to peroxisomes via the ER, PEX16 might be required for trafficking of ssPEX3-GFP from the ER to peroxisomes. Interestingly, the import rates of PEX3-GFP and PMP34 did not decrease upon depletion of *PEX16* expression but, rather, showed significant increase compared to control (Fig. 6D).

### Exogenously expressed PEX16 slows down PEX3 and PMP34 import rates

One explanation for the increase in PEX3-GFP and PMP34-GFP import rates into peroxisomes in cells depleted of PEX16 expression (Fig. 6D) is that these PMPs may be imported into peroxisomes by both the group I and group II pathways. Previously, we reported that ER-localized PEX16 was able to recruit both PEX3-GFP and PMP34-GFP to the ER (Kim et al., 2006). Here, we found that most of the newly synthesized PEX16 initially targeted to the ER before being routed to peroxisomes (Fig. 4). Hence, it is possible that newly synthesized PEX16 is diverting newly synthesized PEX3-GFP and PMP34-GFP to the ER before being transported to peroxisomes. Thus, depleting the cells of PEX16 by using siRNA reduces the group I pathway, resulting in group II pathway to compensate. Since the overall import rate of the group II pathway is faster than that of the group I pathway, the loss of the ER pathways might be causing the observed increase in the import rate of the two PMPs (Fig. 6D).

To test this possibility, we measured the import rate of both PMP-GFP proteins in cells co-transfected with PEX16-mCerulean or mCerulean alone (control). As shown in Fig. 6E, the ssPEX3-GFP apparent import rate was found unaffected by PEX16-mCerulean overexpression compared to control cells. However, the import rates of PMP34-GFP and PEX3-GFP co-transfected with PEX16-mCerulean were about half of those co-transfected with mCerulean alone (*P*<0.005). This decrease in rates was not due to an import competition between PEX16-mCerulean and PMP-GFPs because the import rate of PEX16-GFP did not change when co-expressed with PMP34-mCerulean (Fig. 6E). To further test the hypothesis that the increasing PEX16 expression (via PEX16-mCerulean expression) promotes the group I pathway for PMPs, we examined whether the rate of PEX3-GFP import inversely correlated with PEX16 expression. Indeed, we found that, as the PEX16:PEX3 expression ratio increased, the peroxisomal import rate of PEX3-GFP decreased (Fig. 6F). This difference in rates in cells overexpressing PEX16-mCerulean was not due to a change in peroxisome number because the exogenous expression of PEX16 or PEX3-GFP had no effect on peroxisome numbers (supplementary material Fig. S3G,H). Together with the PEX16 depletion data, our results suggest that PMP34 and PEX3 traffic to peroxisomes by using both group I and group II pathways.

## DISCUSSION

We have demonstrated that the ER is directly involved in maintaining peroxisomes in normal mammalian cells. Using an ER-targeting signal sequence on a peroxisomal membrane protein ssPEX3, we show that it targets to the ER prior to being relocalized to pre-existing peroxisomes. By comparing its peroxisome import kinetics to those of other PMPs, we demonstrate that exogenously expressed PMPs are targeted to peroxisomes via both the group I and group II import pathways. The exception is PEX16, which targets to peroxisomes exclusively via the group I import pathway (via the ER).

The direct evidence that ssPEX3-GFP first targets to the ER before being routed to peroxisomes is the efficient cleavage (>90%) of its preprolactin signal sequence. It has been difficult to observe ssPEX3-GFP on the ER unless it was very highly expressed (Fig. 1E). One interpretation is that ssPEX3-GFP is efficiently transported to peroxisomes after targeting to the ER. Alternatively, it may be released to the cytosol upon the cleavage of its signal sequence at the ER. For example, a small percentage of the ER lumen protein calreticulin is found in the cytosol due to its aborted translocation after the cleavage of its signal sequence (Shaffer et al., 2005). However, we believe that ssPEX3-GFP is efficiently inserted into the ER bilayer and then targeted to peroxisomes after the cleavage of its signal sequence for three reasons. First, the signal sequence of preprolactin has been shown to completely translocate several chimera proteins, including calreticulin, in a highly efficient manner across the ER membrane. Replacing the signal sequence of calreticulin with that of preprolactin greatly reduced the cytosolic localization of calreticulin by preventing the abortion of its translocation into the ER (Shaffer et al., 2005). Second, PEX16, which is transported to peroxisomes from the ER, shows a similar cellular localization profile to that of ssPEX3-GFP at both low and high expression levels (Kim et al., 2006), and also shows a similar import rate (Fig. 4C). Third, if ssPEX3-GFP is efficiently released from the ER into the cytosol then, in the absence of peroxisomes, ssPEX3-GFP should be localized to the cytosol or mitochondria much like the wild type PEX3-GFP. However, we observe ssPEX3-GFP on the ER in cells without pre-existing peroxisomes (Fig. 3; Fig. 5). Similarly, we find that the import kinetics of ssPEX3-GFP are significantly different from those of PEX3-GFP, suggesting a difference in their targeting mechanism. Together these results support the model that ssPEX3-GFP is transported to pre-existing peroxisomes from the ER.

In this study, we have found the import rate of PEX16-GFP to be similar to ssPEX3-GFP, whereas that of PEX3-GFP is comparable to PMP34-GFP (Fig. 4C). The difference in the import rate of PEX16 and ssPEX3 to that of PEX3 and PMP34 is unlikely to be due to differences in their membrane topologies because the faster imported PMP34 contains six transmembrane domains whereas PEX16 possesses only two, and PEX3 and ssPEX3 possess only one transmembrane domain each (Honsho and Fujiki, 2001; Honsho et al., 2002). Similarly, the rate difference between wild-type PEX3 and ER-targeting ssPEX3 is unlikely to be the result of the processing of the signal sequence on ssPEX3, because the removal of the signal sequence is rapid and occurs during translation (Hortin and Boime, 1980; Ibrahimi, 1987; Jungnickel and Rapoport, 1995). Nor is the disparity due to extra residues on the N-terminal end of ssPEX3, because we have found that the import rate of the non-ER targeting construct NssPEX3-GFP is similar to that of PEX3-GFP (data not shown). Hence, we suggest that two different import rates represent two distinct mechanisms of importing peroxisomal proteins to peroxisomes. Given that exogenously expressed PEX3 can target directly to peroxisomes *in vitro* (Matsuzaki and Fujiki, 2008), we propose that the majority of the exogenously expressed PEX3 and PMP34 is directly targeted to peroxisomes (via the group II pathway), whereas PEX16 and ssPEX3 are targeted via the group I pathway.

However, a significant fraction of exogenously expressed PEX3 and PMP34 was targeted to peroxisomes in a PEX16-dependent manner. Depleting endogenous PEX16 expression significantly increased the import rates of the overexpressed PEX3-GFP and PMP34-GFP into peroxisomes (Fig. 6B–D). Conversely, co-expression with PEX16-mCerulean decreased import rates of PEX3-GFP and PMP34-GFP (Fig. 6E) in a PEX16-concentration-dependent manner (Fig. 6F). Because PEX16 is targeted to peroxisomes via the group I pathway, this implies that some of the newly synthesized PEX3 and PMP34 are targeted to peroxisomes via the group I pathway, and the measured peroxisomal import rate of exogenously expressed PEX3 and PMP34 (Fig. 4) is the combination of group I and II pathways.

Although our observations suggest that both PEX3 and PMP34 are able to target to peroxisomes via two pathways, it is not clear whether both pathways are used at endogenous levels or whether one pathway dominates over the other. It is possible that dual targeting pathways for PEX3 and PMP34 are a result of their overexpression. High expression levels of these PMPs may overwhelm one pathway and ‘spill over’ into the other pathway. One possibility is that the group II is the preferred pathway. In this scenario, the predominate pathway is that of direct targeting of PMPs to peroxisomes and the ER pathway is compensating for excess PMP expression. Alternatively, the PEX16-dependent ER pathway (group I pathway) could be predominant pathway, whereas the group II pathway serves as an overflow. Of the two, our data supports the latter scenario. This is based on our observation that the endogenous level of PEX16 is sufficient to slow down the import rates of overexpressed PEX3-GFP and PMP34-GFP (Fig. 6B–D). If the ER pathway was the secondary pathway, knockdown of endogenous PEX16 should not result in an increase of PEX3 and PMP34 import rates. In addition, we find that PEX16 overexpression affects the import rates of these proteins in a concentration-dependent manner (Fig. 6E,F).

Another possible explanation for the changes in PMP import rates upon PEX16 overexpression or depletion is that there may be a common factor that is shared by both pathways. If this common factor or chaperone is involved in both the group I and II pathways, then overexpressing PMPs and PEX16 at the same time might saturate it, thus resulting in a decrease in import rates for both PMPs and PEX16. However, this is unlikely because co-expression of PMP34 and PEX16 did not slow down PEX16 import but only slowed down PMP34 import (Fig. 6E). Therefore, our data suggests that PMPs favour the PEX16-dependent group I pathway over the group II pathway; and we propose that the group I pathway is the default import pathway for PMP import to peroxisomes.

**Fig. 7.**
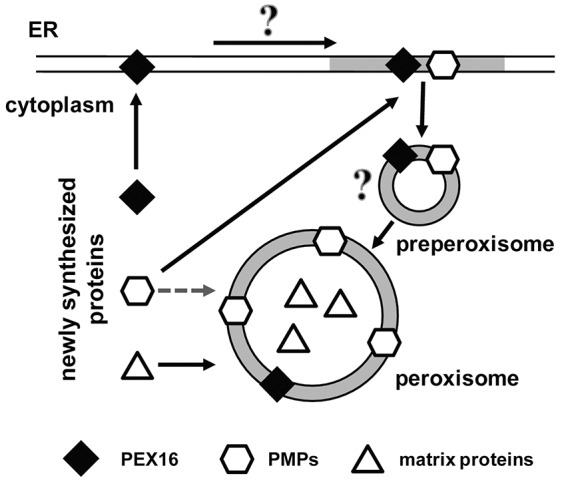
**Model of PMP import into peroxisomes within mammalian cells.** PMPs can be targeted to peroxisomes via two distinct pathways: the group I pathway through which PMPs initially target to the ER before being routed to peroxisomes; or the group II pathway through which PMPs are directly imported to peroxisomes. The pathway utilized by the PMP depends on the level of PEX16 in the ER, which is co-translationally targeted to the ER. On the ER, PEX16 can recruit other PMPs to the ER where they are rapidly transported to pre-existing peroxisomes. The mechanism of the transport between ER to peroxisomes has yet to be determined. However, based on studies using yeast and plant cells, PEX16 and PMPs may accumulate in a specialized domain on the ER that is enriched for PMPs and peroxisomal lipids (grey membrane) in order to generate pre-peroxisomal vesicles. Under conditions where PMPs are in excess compared to PEX16, such as an ectopic expression of PMPs, they can also target directly to peroxisomes via the group II pathway. Matrix proteins (triangles) are directly targeted to mature peroxisomes from the cytoplasm.

Generalizing these findings, we put forward the following model for the maintenance of peroxisomes in the mammalian cell (Fig. 7). We propose that most PMPs are targeted to peroxisomes via two distinct pathways, group I and group II, and the extent to which each pathway is used is dependent on the ratio of newly synthesized PEX16 to PMP. We suggest that the group I pathway is the dominant one, meaning that most PMPs are targeted initially to the ER by PEX16 before being routed to peroxisomes. Significantly increasing the expression of PMPs can saturate the ER recruitment pathway because of the limited amount of PEX16, in which case they would be imported directly to peroxisomes via the group II pathway. Although we depict a small vesicle between the ER and peroxisomes (Fig. 7), how exactly the two organelles communicate is not known in the mammalian system.

Our model shares some similarities to current models of PMP import into *S. cerevisiae*. PMPs in *S. cerevisiae* have been reported to import via both the group I and II pathways (van der Zand et al., 2010; Thoms et al., 2012; Huybrechts et al., 2009). However, unlike in the mammalian system, where the ER provides new PMPs to pre-existing peroxisomes, the ER in *S. cerevisiae* does not provide new PMPs to pre-existing peroxisomes but, rather, is involved in forming pre-peroxisomal vesicles that fuse to form new peroxisomes that originate at the ER (Titorenko et al., 2000; van der Zand et al., 2012). In *S. cerevisiae*, PMPs are targeted to the ER exclusively for the *de novo* biogenesis of peroxisomes, whereas the group II targeting mechanism is only involved in the maintenance of pre-existing peroxisomes. This mechanism differs from our model of peroxisome maintenance in the mammalian system, in which the ER constitutively provides PMPs to peroxisomes. Furthermore, we suggest in our model that, during high peroxisome proliferation events – such as activation of nuclear peroxisome-proliferator-activated receptors (PPARs), when peroxisome numbers are rapidly increased four- to tenfold in a very short time frame (Reddy, 2004) – any excess of synthesized PMP is likely to be targeted via the group II pathway. However, even in high peroxisome proliferation conditions, the ER is probably heavily involved in maintaining peroxisomes by providing lipids and key PMPs, such as PEX16, to the dividing peroxisomes. In fact, PEX16 expression is upregulated upon the activation of PPARγ (Karnik et al., 2009). Since we find that the majority of the newly synthesized PEX16 is first targeted to the ER, we suggest that the ER plays an essential role in facilitating peroxisome proliferation by providing those PMPs that are required for peroxisome proliferation. However, under conditions where the total amount of PMPs exceed the capacity of PEX16, PMPs can also be targeted directly to peroxisomes via the group II pathway.

In summary, we show two distinct routes of PMP import into pre-existing peroxisomes in mammalian cells: direct targeting to peroxisomes and indirect targeting via the ER. In addition, we show evidence that the ER constitutively provides at least two very distinct PMPs, PEX3 and PMP34, to pre-existing peroxisomes, and that this mechanism is dependent on PEX16.

## MATERIALS AND METHODS

### Cell lines and reagents

HeLa human epithelial carcinoma cells were obtained from American Type Culture Collection (ATCC). PBD400-T1 cells (South et al., 2000) were a gift from Stephen J. Gould (Johns Hopkins University, Baltimore). GM06231 cells were purchased from NIGMS Human Genetic Mutant Cell Repository, Coriell Institute for Medical Research, Camden, NJ, USA. Rabbit anti-GFP was a gift from Ramanujan Hegde (MRC, LMB, Cambridge, UK).

### Plasmid constructs

The construction of plasmids encoding PEX16-GFP, PMP34-GFP, UB-GFP-SKL, PEX16-mCerulean, mRFP-SKL, ssRFP-KDEL PEX3-GFP and cDNA3-PEX3–3×myc and Venus-Omp25TM has been described previously (Kim et al., 2006; South et al., 2000; Wang et al., 2012). Plasmid construct strategies used and the primer sequences are available from the authors upon request.

### Culture conditions and transfections

HeLa cells were cultured in Dulbecco's modified Eagle's medium (DMEM)/High without L-glutamine (Thermo Scientific HyClone) supplemented with 10% fetal bovine serum (FBS; GIBCO/Invitrogen) and 2 mM L-glutamine (Thermo Scientific HyClone). Cells were grown at 37°C in 5% CO_2_ in humidified air. Transient transfections were performed using Lipofectamine™ 2000 reagent according to the manufacturer's standard protocol (Invitrogen). PBD400-T1 or GM06231 cells were cultured in freshly prepared DMEM supplemented with 15% of FBS and 2 mM L-glutamine, and transfected by electroporation (Amaxa Nucleofection, program V-13) using the Amaxa Basic Nucleofector kit for primary fibroblasts (Lonza).

For live cell microscopy, cells were seeded onto four-well (Lab-Tek™, Nunc) chambered coverglasses and cultured under standard conditions for 24 hours prior to transfection. Cells were then imaged at the indicated time in CO_2_ independent medium (GIBCO/Invitrogen) at 37°C. For the induction of Tet-On system, pTET-On-ssPEX3-GFP transfected into HeLa Tet-On advanced cells was induced using 0.1 µg/ml of doxycycline (Sigma) as suggested by the manufacturer (Clontech).

### Fluorescence microscopy in living cells; time-lapse experiments

All fluorescence images were acquired by using a Zeiss LSM 710 laser scanning confocal microscope. For analysis of protein import into peroxisomes the images were acquired by using a 40×1.3NAPlan-Neofluar oil-immersion objective with LSM710 ZEN2009 software. The GFP signal was excited (in presence of mCerulean) by using a 488 nm Argon laser with a 493–565 nm or 515–565 nm bandpass filter. RFP was excited by using a 561 nm diode laser with a 600–700 nm bandpass. mCerulean images were acquired by using a 405 nm diode laser with a 450–495 nm bandpass filter. PARFP-SKL was photoactivated with the 405 nm diode laser and imaged by using the RFP setting. Brightness and contrast in representative images shown in the article were adjusted on ImageJ program to improve visibility, with the same brightness and contrast adjustments for all frames of time-lapse images. All analysis was performed on the original (unadjusted) 8-bit images.

### Quantification of import rate of peroxisomal protein into peroxisomes

The quantitative analysis was performed using ImageJ program (Wayne S. Rasband, ImageJ, NIH, http://rsb.info.nih.gov/ij/). The maximal area of each cell enriched with GFP signal, excluding nucleus, was selected as region of interest (ROI). Peroxisomes within the cell were identified by masking the signal of ubiquitin fused to red fluorescent protein tagged to the amino acids Ser, Lys and Leu (UB-RFP-SKL) by using a threshold. The threshold was chosen to maximize the number of peroxisomal punctuate structures within an area of 0.1–1 µm^2^. The average pixel intensities of the background of GFP (C_G_) and RFP (C_R_) fluorescent signals were quantified by averaging the signal from ten non-peroxisomal regions distributed evenly throughout the selected ROI in close proximity to peroxisomes. The fluorophor pairs choosen for our studies were monomerized GFP and monomerized RFP. They were selected owing to to their non-overlapping spectra, their monomeric nature and also the similarity in their maturation rate (∼1 hour) (Shaner et al., 2004; Sniegowski et al., 2005).

### Quantification of relative import rates for PMP-GFP and UB-GFP-SKL

HeLa cells were transfected with PMP-GFP and UB-RFP-SKL. The imaging was started 20 hours after transfection. The following criteria were used when selecting cells for analysis: (i) cells that show very low expression of both GFP and RFP in early time frames, (ii) cells with GFP and RFP expression within the dynamic range of the photomultiplier tube (PT) detection and, (iii) cells in which the peroxisomal RFP and GFP expression increased over time without saturating the PT. The calculation of RFP and GFP signals (*P_R_* and *P_G_*, respectively) in peroxisomes was as follows:
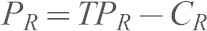
(1a)
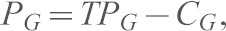
(1b)where *TP_R_* and *TP_G_* are average pixel intensity of RFP and GFP in peroxisomes, respectively, and *C_R_* and *C_G_* are average pixel intensity of RFP and GFP background, respectively.

To ensure the same fluorescence intensity signal units for GFP and RFP, we converted the intensity of GFP expressed in AU of GFP into AU of RFP. For these conversions we used the GFP-RFP-SKL construct, which has GFP and RFP in tandem with a PTS1 at its C-terminus. This construct should give a constant RFP/GFP signal ratio (*r*):
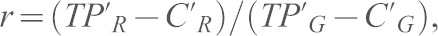
(2a)where 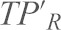 and 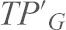 are average pixel intensity of RFP and GFP in peroxisomes, respectively, of the GFP-RFP-SKL construct. 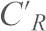 and 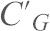 are average pixel intensity of RFP and GFP background, respectively, of the GFP-RFP-SKL construct.

We used this ratio as a coefficient between GFP and RFP signal units. Images of HeLa cells expressing GFP-RFP-SKL were acquired in the same way as described previously, and the ratio was determined each day of the experiment.

The GFP signal units conversion in peroxisomes was calculated as follows:
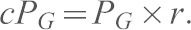
(2b)The fluorescence signal for both GFP (*cP_G_*) and RFP *(P_R_*) protein was plotted against time and fitted with linear regression:
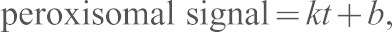
(3)where *k* is the apparent import rate of labeled protein into peroxisomes in arbitrary unit per hour (AU/h), *t* is time in hours and b is an initial signal value of measured fluorescent protein at time zero.

To compare the kinetics of different PMP-GFPs on different days, the import rate of GFP (*k_GFP_*) was normalized to the import rate of RFP (*k_RFP_*) within the same cell. The import ratio of GFP and RFP was designated as the relative rate of import (*k_GFP_/k_RFP_*) and calculated for every cell. The relative rates for all analyzed cells from all independent trials were pooled and presented as a the mean±s.e.m. in Fig. 4C.

### Import rate of PMP-GFPs in cells treated with siRNA and in cells that express PEX16 exogenously

HeLa cells stably expressing RFP-tagged SKL protein (RFP-SKL) were treated with siCNTR or siPEX16 prior to transfection with the plasmid encoding the PMP-GFP protein of interest. The imaging was started 7 hours after transfection. Peroxisomes within the cell were identified by the RFP-SKL signal. The GFP signal in peroxisomes was calculated using Eqn 1b.

The fluorescence signal for PMP-GFP (*P_G_*) was plotted against time and fitted with linear regression (Eqn 3). To make the data comparable between the experiments, the average import rate of siCNTR-treated cells [*k_AV_*_(*siCNTR*)_] in a given experiment was calculated as follows:
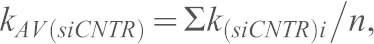
(4)where *k*_(*siCNTR*)*i*_ is the import rate of individually analyzed siCNTR-treated cell in a given experiment and *n* is the number of analyzed siCNTR-treated cells in this experiment. The import rate for each siPEX16-treated cell [*k*_(*siPEX16*)_] was normalized to the average import rate for siCNTR treated cells [*k_AV_*_(*siCNTR*)_] from the same experiment and presented as the relative import rate [*k*_(*siPEX16*)_/*k_AV_*_(*siCNTR*)_]. The relative import rates for all analyzed siPEX16-treated cells from three independent trials (20 cells per trial per treatment) were pooled together and presented as a the mean±s.e.m. in Fig. 5D.

The same measurement and calculation strategy was used in experiments where various PMP-GFP constructs were co-expressed with PEX16-mCerulean or PMP34-mCerulean (Fig. 5E). Cells co-expressing mCerulean instead of PEX16-mCerulean or PMP34-mCerulean were used as a control. For a given PMP-GFP construct, its import rate [*k*_(*PMP-GFP*)_] for each individual cell co-expressing PEX16-mCerulean or PMP34-mCerulean was normalized to the average import rate for cells co-expressing mCerulean control [*k_AV_*_(*CNTR*)_] from the same experiment. The resulting relative import rates [*k*_(*PMP-GFP*)_/*k_AV_*_(*CNTR*)_] for all analyzed cells from three independent trials (20 cells per trial per treatment) were pooled together and presented as the mean ± s.e.m. in Fig. 5E.

### Distribution of PMP-GFP signal in the cell at fixed time points

HeLa cells stably expressing RFP-SKL protein were treated with siCNTR or siPEX16 prior to transfecting the cells with the plasmid encoding the PMP-GFP protein of interest as described below. The GFP signal inside peroxisome was determined by using RFP-SKL for peroxisome detection. Plasmids encoding ssPEX3-GFP, PEX3-GFP or PMP34-GFP were transfected on the third day after the first siRNA treatment and the images were acquired 20 hours later (on the fourth day). The average GFP fluorescent intensity within peroxisomes (I_per._) and in the whole cells (I_cell_) was determined 20 hours after transfection. PMP-GFP distribution was presented as the intensity ratio (I_per._:I_cell_) (Fig. 5A–C). In each of the three performed experiments, the peroxisomal import rate of PMP-GFP protein was determined for at least 50 cells for every treatment (150 cells in total).

### Western blots

For western blot analysis, cells grown in six-well plates were washed twice with PBS and lysed in 100 µl of Lysis buffer (0.1 M Tris-HCl, pH 9; 1% SDS). Lysate was collected and incubated at 100°C for 15 minutes with intervals of vigorous vortexing to shred the genomic DNA. Protein concentration was determined with a BCA protein assay kit (Novagen). Lysates containing 10 µg of total protein was analyzed separated in 8% SDS-PAGE (Laemmli, 1970), transferred to PVDF membrane, and membrane was probed for GFP using a rabbit anti-GFP antibody at dilution 1∶5000 and subsequently goat anti-rabbit antibody conjugated to horseradish peroxidase at dilution 1∶15,000 (Cedarlane Laboratories). The blot was visualized using an enhanced chemiluminescence kit from Pierce.

### Immunofluorescence assay

Immunofluorescence was performed as previously described (Kim et al., 2006). Briefly, the cells were fixed with 4% paraformaldehyde and permeablized using 0.1% TX-100 in PBS. Catalase was probed using a rabbit polyclonal anti-catalase antibody at dilution 1∶2000, and GFP using a mouse anti-GFP antibody at dilution 1∶2000. Alexa-Fluor-633 antibody (Invitrogen) was used for visualization, as indicated, at dilution 1∶1000.

### Knockdown assay

PEX16 expression in HeLa cells was depleted using the siRNA sense sequence 5′-UGACGGGAUCCUACGGAAGdTdT-3′ (Shanhai GenePharma Co.) as previously described (Fang et al., 2004; Kim et al., 2008). Briefly, HeLa cells constantly expressing RFP-SKL were transfected with either siRNA PEX16 or non-targeting control twice in a 24-hour interval using Lipofectamine™ 2000 reagent.

For rate determination experiments, pssPEX3-GFP, pPEX3-GFP or pPMP34-GFP were transfected on the 3rd day after the first siRNA treatment and the time-lapse imaging experiment was started 7 hours later (3rd day). Cells treated with PEX16 siRNA and siCNTR control RNA were tested in parallel on 20 different fields each. In each of the three performed experiments, the peroxisomal import rate of GFP-labeled protein was determined in at least 20 cells for every treatment (in total about 60 cells). The RFP-SKL was used to determine the GFP signal in peroxisomes as described above.

The effect of siRNA on the relative transcription level of pex16 was determined with real-time PCR using Mastercycler ep realplex software (Eppendorf) for analysis using SYBR Green PCR Master Mix (ABI). Total RNA was isolated using SV Total RNA Isolation System (Promega), and Go Script Reverse Transcriptase (Promega) with specific primers for pex16 5′-aaaagtcgacccccaactgtagaagtagattttc-3′ and for β-actin 5′-aatgtcacgcacgatttccc-3′ (reference gene) was used. Each sample was measured in triplicate.

## Supplementary Material

Supplementary Material

## References

[b2] Brocard C. B. Boucher K. K. Jedeszko C. Kim P. K. Walton P. A. (2005). Requirement for microtubules and dynein motors in the earliest stages of peroxisome biogenesis. Traffic 6, 386–395 10.1111/j.1600-0854.2005.00283.x 15813749

[b5] Fang Y. Morrell J. C. Jones J. M. Gould S. J. (2004). PEX3 functions as a PEX19 docking factor in the import of class I peroxisomal membrane proteins. J. Cell Biol. 164, 863–875 10.1083/jcb.200311131 15007061 PMC2172291

[b6] Hoepfner D. Schildknegt D. Braakman I. Philippsen P. Tabak H. F. (2005). Contribution of the endoplasmic reticulum to peroxisome formation. Cell 122, 85–95 10.1016/j.cell.2005.04.025 16009135

[b8] Honsho M. Tamura S. Shimozawa N. Suzuki Y. Kondo N. Fujiki Y. (1998). Mutation in PEX16 is causal in the peroxisome-deficient Zellweger syndrome of complementation group D. *Am.* J. Hum. Genet. 63, 1622–1630 10.1086/302161 PMC1377633 9837814

[b9] Honsho M. Hiroshige T. Fujiki Y. (2002). The membrane biogenesis peroxin Pex16p. Topogenesis and functional roles in peroxisomal membrane assembly. J. Biol. Chem. 277, 44513–44524 10.1074/jbc.M206139200 12223482

[b10] Hortin G. Boime I. (1980). Pre-prolactin accumulates in rat pituitary cells incubated with a threonine analog. J. Biol. Chem. 255, 7051–7054 6771282

[b11] Huybrechts S. J. Van Veldhoven P. P. Brees C. Mannaerts G. P. Los G. V. Fransen M. (2009). Peroxisome dynamics in cultured mammalian cells. Traffic 10, 1722–1733 10.1111/j.1600-0854.2009.00970.x 19719477

[b12] Ibrahimi I. (1987). Dithiothreitol and the translocation of preprolactin across mammalian endoplasmic reticulum. J. Cell Biol. 105, 1555–1560 10.1083/jcb.105.4.1555 3667690 PMC2114654

[b13] Jungnickel B. Rapoport T. A. (1995). A posttargeting signal sequence recognition event in the endoplasmic reticulum membrane. Cell 82, 261–270 10.1016/0092-8674(95)90313-5 7628015

[b14] Karnik P. Tekeste Z. McCormick T. S. Gilliam A. C. Price V. H. Cooper K. D. Mirmirani P. (2009). Hair follicle stem cell-specific PPARgamma deletion causes scarring alopecia. J. Invest. Dermatol. 129, 1243–1257 10.1038/jid.2008.369 19052558 PMC3130601

[b15] Kim P. K. Mullen R. T. Schumann U. Lippincott-Schwartz J. (2006). The origin and maintenance of mammalian peroxisomes involves a de novo PEX16-dependent pathway from the ER. J. Cell Biol. 173, 521–532 10.1083/jcb.200601036 16717127 PMC2063862

[b16] Kim P. K. Hailey D. W. Mullen R. T. Lippincott-Schwartz J. (2008). Ubiquitin signals autophagic degradation of cytosolic proteins and peroxisomes. Proc. Natl. Acad. Sci. USA 105, 20567–20574 10.1073/pnas.0810611105 19074260 PMC2602605

[b17] Kragt A. Voorn-Brouwer T. van den Berg M. Distel B. (2005). Endoplasmic reticulum-directed Pex3p routes to peroxisomes and restores peroxisome formation in a Saccharomyces cerevisiae pex3Delta strain. J. Biol. Chem. 280, 34350–34357 10.1074/jbc.M505432200 16100114

[b18] Laemmli U. K. (1970). Cleavage of structural proteins during the assembly of the head of bacteriophage T4. Nature 227, 680–685 10.1038/227680a0 5432063

[b20] Lazarow P. B. Fujiki Y. (1985). Biogenesis of peroxisomes. Annu. Rev. Cell Biol. 1, 489–530 10.1146/annurev.cb.01.110185.002421 3916321

[b21] Lodish H. F. (1988). Transport of secretory and membrane glycoproteins from the rough endoplasmic reticulum to the Golgi. A rate-limiting step in protein maturation and secretion. J. Biol. Chem. 263, 2107–2110 3276683

[b22] Ma C. Agrawal G. Subramani S. (2011). Peroxisome assembly: matrix and membrane protein biogenesis. J. Cell Biol. 193, 7–16 10.1083/jcb.201010022 21464226 PMC3082194

[b23] Matsuzaki T. Fujiki Y. (2008). The peroxisomal membrane protein import receptor Pex3p is directly transported to peroxisomes by a novel Pex19p- and Pex16p-dependent pathway. J. Cell Biol. 183, 1275–1286 10.1083/jcb.200806062 19114594 PMC2606968

[b24] Menendez-Benito V. van Deventer S. J. Jimenez-Garcia V. Roy-Luzarraga M. van Leeuwen F. Neefjes J. (2013). Spatiotemporal analysis of organelle and macromolecular complex inheritance. Proc. Natl. Acad. Sci. USA 110, 175–180 10.1073/pnas.1207424110 23248297 PMC3538235

[b25] Motley A. M. Hettema E. H. (2007). Yeast peroxisomes multiply by growth and division. J. Cell Biol. 178, 399–410 10.1083/jcb.200702167 17646399 PMC2064844

[b26] Nuttall J. M. Motley A. Hettema E. H. (2011). Peroxisome biogenesis: recent advances. Curr. Opin. Cell Biol. 23, 421–426 10.1016/j.ceb.2011.05.005 21689915

[b27] Reddy J. K. (2004). Peroxisome proliferators and peroxisome proliferator-activated receptor alpha: biotic and xenobiotic sensing. Am. J. Pathol. 164, 2305–2321 10.1016/S0002-9440(10)63787-X 15161663 PMC1615758

[b28] Rucktäschel R. Girzalsky W. Erdmann R. (2011). Protein import machineries of peroxisomes. Biochim. Biophys. Acta 1808, 892–900 10.1016/j.bbamem.2010.07.020 20659419

[b29] Sacksteder K. A. Jones J. M. South S. T. Li X. Liu Y. Gould S. J. (2000). PEX19 binds multiple peroxisomal membrane proteins, is predominantly cytoplasmic, and is required for peroxisome membrane synthesis. J. Cell Biol. 148, 931–944 10.1083/jcb.148.5.931 10704444 PMC2174547

[b30] Santos M. J. Imanaka T. Shio H. Small G. M. Lazarow P. B. (1988). Peroxisomal membrane ghosts in Zellweger syndrome—aberrant organelle assembly. Science 239, 1536–1538 10.1126/science.3281254 3281254

[b31] Schmidt F. Dietrich D. Eylenstein R. Groemping Y. Stehle T. Dodt G. (2012). The role of conserved PEX3 regions in PEX19-binding and peroxisome biogenesis. Traffic 13, 1244–1260 10.1111/j.1600-0854.2012.01380.x 22624858

[b32] Shaffer K. L. Sharma A. Snapp E. L. Hegde R. S. (2005). Regulation of protein compartmentalization expands the diversity of protein function. Dev. Cell 9, 545–554 10.1016/j.devcel.2005.09.001 16198296

[b33] Shaner N. C. Campbell R. E. Steinbach P. A. Giepmans B. N. Palmer A. E. Tsien R. Y. (2004). Improved monomeric red, orange and yellow fluorescent proteins derived from Discosoma sp. red fluorescent protein. Nat. Biotechnol. 22, 1567–1572 10.1038/nbt1037 15558047

[b35] Sniegowski J. A. Lappe J. W. Patel H. N. Huffman H. A. Wachter R. M. (2005). Base catalysis of chromophore formation in Arg96 and Glu222 variants of green fluorescent protein. J. Biol. Chem. 280, 26248–26255 10.1074/jbc.M412327200 15888441

[b36] Soukupova M. Sprenger C. Gorgas K. Kunau W. H. Dodt G. (1999). Identification and characterization of the human peroxin PEX3. Eur. J. Cell Biol. 78, 357–374 10.1016/S0171-9335(99)80078-8 10430017

[b37] South S. T. Sacksteder K. A. Li X. Liu Y. Gould S. J. (2000). Inhibitors of COPI and COPII do not block PEX3-mediated peroxisome synthesis. J. Cell Biol. 149, 1345–1360 10.1083/jcb.149.7.1345 10871277 PMC2175136

[b38] South S. T. Baumgart E. Gould S. J. (2001). Inactivation of the endoplasmic reticulum protein translocation factor, Sec61p, or its homolog, Ssh1p, does not affect peroxisome biogenesis. Proc. Natl. Acad. Sci. USA 98, 12027–12031 10.1073/pnas.221289498 11593013 PMC59761

[b39] Steinberg S. J. Dodt G. Raymond G. V. Braverman N. E. Moser A. B. Moser H. W. (2006). Peroxisome biogenesis disorders. Biochim. Biophys. Acta 1763, 1733–1748 10.1016/j.bbamcr.2006.09.010 17055079

[b40] Tam Y. Y. Fagarasanu A. Fagarasanu M. Rachubinski R. A. (2005). Pex3p initiates the formation of a preperoxisomal compartment from a subdomain of the endoplasmic reticulum in Saccharomyces cerevisiae. J. Biol. Chem. 280, 34933–34939 10.1074/jbc.M506208200 16087670

[b41] Theodoulou F. L. Bernhardt K. Linka N. Baker A. (2013). Peroxisome membrane proteins: multiple trafficking routes and multiple functions? Biochem. J. 451, 345–352 10.1042/BJ20130078 23581405

[b42] Thoms S. Harms I. Kalies K. U. Gärtner J. (2012). Peroxisome formation requires the endoplasmic reticulum channel protein Sec61. Traffic 13, 599–609 10.1111/j.1600-0854.2011.01324.x 22212716

[b43] Titorenko V. I. Rachubinski R. A. (2001). The life cycle of the peroxisome. Nat. Rev. Mol. Cell Biol. 2, 357–368 10.1038/35073063 11331910

[b44] Titorenko V. I. Chan H. Rachubinski R. A. (2000). Fusion of small peroxisomal vesicles in vitro reconstructs an early step in the in vivo multistep peroxisome assembly pathway of Yarrowia lipolytica. J. Cell Biol. 148, 29–44 10.1083/jcb.148.1.29 10629216 PMC2156211

[b45] Toro A. A. Araya C. A. Córdova G. J. Arredondo C. A. Cárdenas H. G. Moreno R. E. Venegas A. Koenig C. S. Cancino J. Gonzalez A. (2009). Pex3p-dependent peroxisomal biogenesis initiates in the endoplasmic reticulum of human fibroblasts. J. Cell. Biochem. 107, 1083–1096 10.1002/jcb.22210 19479899

[b46] van den Bosch H. Schutgens R. B. Wanders R. J. Tager J. M. (1992). Biochemistry of peroxisomes. Annu. Rev. Biochem. 61, 157–197 10.1146/annurev.bi.61.070192.001105 1353950

[b47] van der Zand A. Braakman I. Tabak H. F. (2010). Peroxisomal membrane proteins insert into the endoplasmic reticulum. Mol. Biol. Cell 21, 2057–2065 10.1091/mbc.E10-02-0082 20427571 PMC2883949

[b48] van der Zand A. Gent J. Braakman I. Tabak H. F. (2012). Biochemically distinct vesicles from the endoplasmic reticulum fuse to form peroxisomes. Cell 149, 397–409 10.1016/j.cell.2012.01.054 22500805

[b49] von Heijne G. (1985). Signal sequences. The limits of variation. J. Mol. Biol. 184, 99–105 10.1016/0022-2836(85)90046-4 4032478

[b50] Wang Y. Nartiss Y. Steipe B. McQuibban G. A. Kim P. K. (2012). ROS-induced mitochondrial depolarization initiates PARK2/PARKIN-dependent mitochondrial degradation by autophagy. Autophagy 8, 1462–1476 10.4161/auto.21211 22889933

[b52] Zaal K. J. Smith C. L. Polishchuk R. S. Altan N. Cole N. B. Ellenberg J. Hirschberg K. Presley J. F. Roberts T. H. Siggia E. (1999). Golgi membranes are absorbed into and reemerge from the ER during mitosis. Cell 99, 589–601 10.1016/S0092-8674(00)81548-2 10612395

