# Supplementary material for: PEX16 contributes to peroxisome maintenance by constantly trafficking PEX3 via the ER
Source: J Cell Sci. 2014 Sep 1;127(17):3675–86. doi: 10.1242/jcs.146282 (PMC4172262; doi:10.1242/jcs.146282)
Supplement: Supplementary Material [file supp_127_17_3675__index.html]

PEX16 contributes to peroxisome maintenance by constantly trafficking PEX3 via the ER — Supplementary Material 

# PEX16 contributes to peroxisome maintenance by constantly trafficking PEX3 via the ER

## JCS146282 Supplementary Material

**Files in this Data Supplement:**

- **Supplementary Material**
