# Supplementary material for: PEX16 contributes to peroxisome maintenance by constantly trafficking PEX3 via the ER
Source: J Cell Sci. 2014 Sep 1;127(17):3675–86. doi: 10.1242/jcs.146282 (PMC4172262; doi:10.1242/jcs.146282)
Supplement: Supplementary Material [file supp_127.17.3675_JCS146282.pdf]

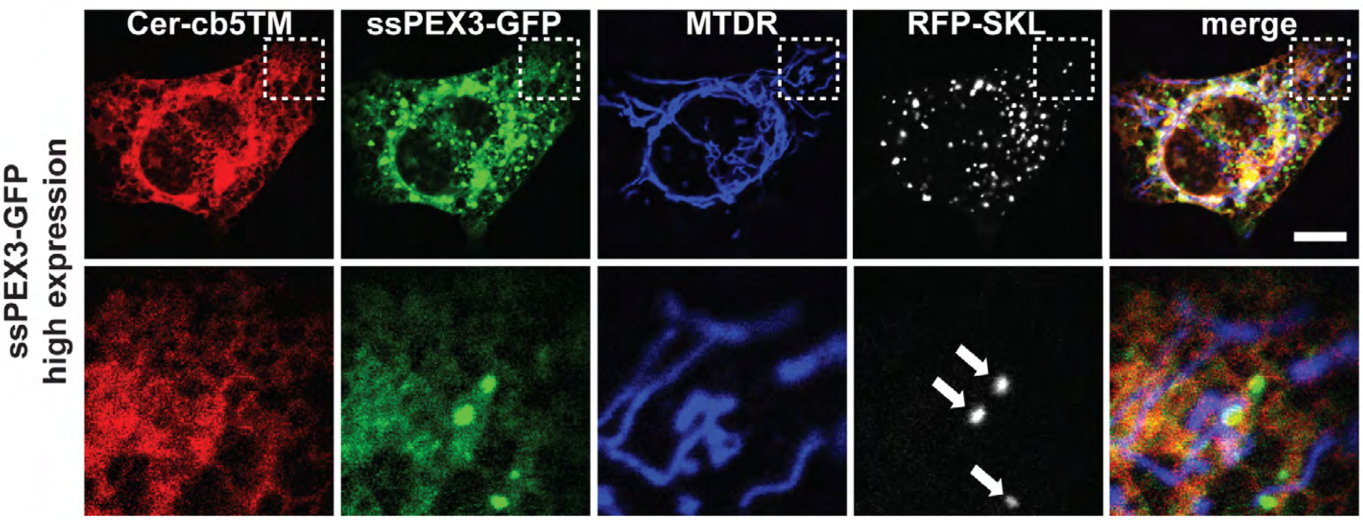

Fig. S1. At high expression level, ssPEX3-GFP co-localized with ER and peroxisome markers but not with a mitochondrial marker. Representative confocal fluorescence microscopy images of high ssPEX3-GFP expressing living cell. HeLa cells stably expressing RFP-SKL were co- transfected with pssPEX3-GFP and pmCerulean-cb5(TM) (an ER marker). On the day after transfection, the cells were exposed to 50 μM of MitoTracker Deep Red (MTDR: a mitochondria marker) 20 mins before imaging. Shown is each marker in pseudo colours: ER (red), ssPEX3-GFP (green), Mitochondria (cyan) and peroxisomes (white), and merge of all four panels on the left. Dashed square indicates the magnified region shown on the corresponding bottom panels. Arrows indicate peroxisomes. Scale bar is 10μm.

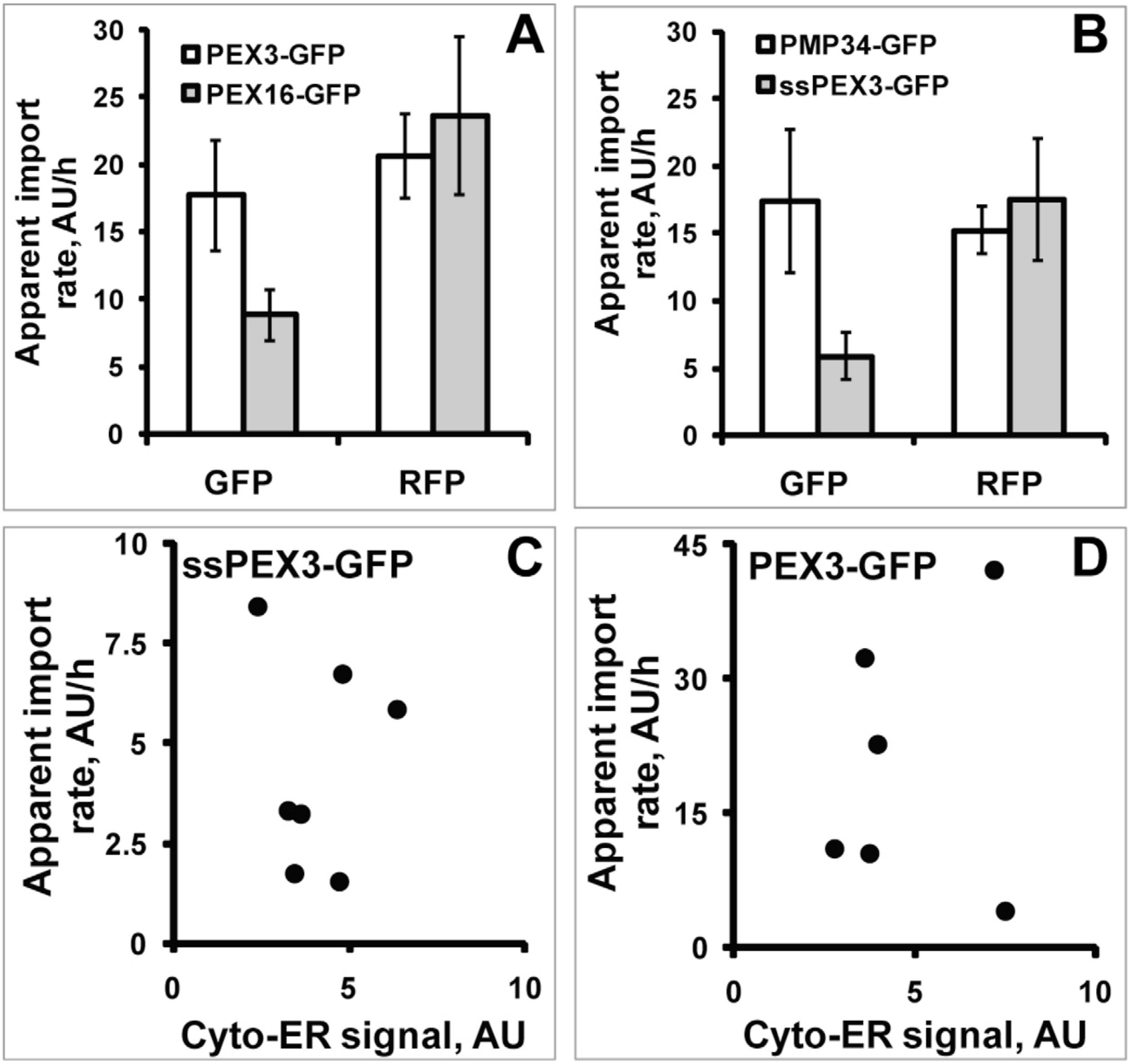

Fig. S2. (A,B) The apparent import rate of UB-RFP-SKL is independent of GFP-fused PMPs. (A) HeLa cells co-transfected with UB-RFP-SKL/PEX3-GFP or UB-RFP- SKL/PEX16-GFP were imaged and analyzed in parallel as described in Fig. 4. The averages of the apparent peroxisomal import rates are plotted versus the corresponding GFP and RFP signals. (B) Same as in (A) but cells were co-transfected with the plasmids encoding for UB-RFP-SKL/ssPEX3-GFP or UB-RFP-SKL/PMP34-GFP. For each pair of PMPs (A or B) images were obtained in parallel in a single time-lapse experiment to ensure same microscope settings so that their apparent import rates can be readily compared. The GFP and RFP signals in peroxisomes were calculated and the apparent import rates were determined. In all cases, the GFP-fused proteins do not change the apparent import rate of UB-RFP-SKL. The error bars are the apparent import rate standard error of the mean. **(C, D) Apparent import rate of GFP-fused protein into peroxisomes is independent of the protein expression level.** Time-lapse experiment of HeLa cells co-expressing ssPEX3-GFP (C) or PEX3-GFP (D) together with UB-RFP-SKL. GFP and RFP fluorescent signals were determined in peroxisomal and non-peroxisomal (cytoplasm-ER) space for each cell (see Materials and methods). The apparent import rate (not normalized against RFP/GFP ratio from GFP-RFP-SKL) for each cell is plotted versus corresponding cytosol and ER signals (cyto-ER). All represented cells were from the same experiment performed on the same day in order to minimize signal variations. Each point on the graphs represents one cell.

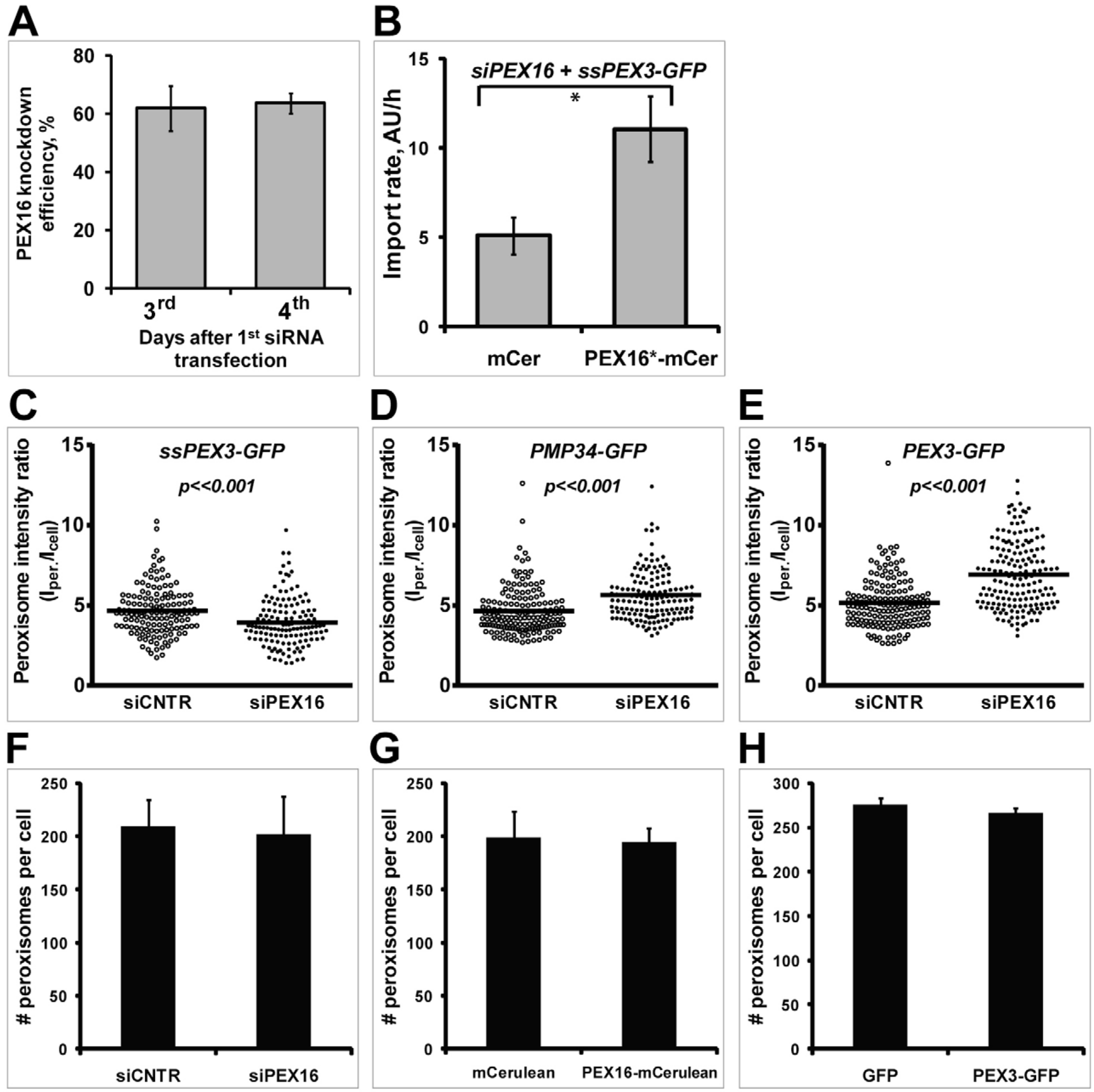

Fig. S3. PEX16 knockdown. (A) Evaluation of PEX16 knockdown efficiency. Level of knockdown was evaluated by transcription efficiency of PEX16 on 3rd and 4th days after transfection using quantitative real-time PCR at the indicated days (see Materials and Methods). Each bar represents the mean ± standard error of the mean of three independent experiments. (B) PEX16 RNAi complementation assay by the co-expression of the siRNA with the siRNA-resistant PEX16 construct (PEX16*-mCerulean). The peroxisomal import rates of ssPEX3-GFP in PEX16-depleted cells exogenously co-expressing ssPEX3-GFP and mCerulean are compared to those cells co- expressing ssPEX3-GFP and PEX16*-mCerulean. In the presence of PEX16*-mCerulean the import rate is approximately doubled, which is in agreement with Fig. 6 (D, E).

(C-E) Scatter plot presentation of results shown in Fig. 6 A-C respectively.

(F-H) PEX16 depletion/overexpression or PEX3 overexpression does not significantly affect peroxisome number in cells. (F) HeLa cells treated with siCNTR or siPEX16 were fixed on 4th day after 1st siRNA treatment. (G) HeLa cells were transfected with plasmid encoding either PEX16-mCerulean or Cerulean-N1 plasmids and incubated for 2 days. (H) HeLa cells were transfected with either pPEX3-GFP or EGFP-N1 plasmids and incubated for 2 days. (F-H) At the indicated time, cells were fixed and probed with rabbit anti-PMP70 antibody followed by staining with goat anti- rabbit ALEXA-633 secondary antibody. The z-stacks were collected and the number of peroxisomes was counted in 100 cells for each treatment using the ImageJ program. The error bars are the peroxisome number standard deviation.
